# Mediterranean Journal of Rheumatology September 2017 Highlights

**DOI:** 10.31138/mjr.28.3.110

**Published:** 2017-09-29

**Authors:** Lazaros I. Sakkas

**Affiliations:** Department of Rheumatology and Clinical Immunology, Faculty of Medicine, School of Health Sciences, University of Thessaly, Larissa, Greece

In this issue of MJR, readers will find educational sources to help them in their clinical practice.

In an editorial,^1^ A. Sandoo reviewed data on endothelial dysfunction in rheumatoid arthritis (RA). RA has a similar cardiovascular disease risk to that of type II diabetes mellitus. Non-invasive assessment of microvessels (endothelium-dependent and endothelium-independent) and large arteries can predict adverse cardiovascular outcome in the general population but this is to be shown in RA.

Tchetina^2^ examines the role of gene expression in RA and how this can help in individualized patient treatment (personalized medicine). High TNFα and /or type I IFN response gene expression may suggest good response to TNF inhibitors and IL-6 blockade whereas low expression of type I IFN response genes may suggest good response to anti-B cell treatment. Elevated serum levels of matrix metalloproteinase -1 (MMP-1) and MMP-3 and high expression of TGFβ correlate with erosive disease. These biomarkers may be taken into account when a practitioner decides on which treatment in each patient with RA.

Magro et al.^3^ reviewed the evidence of vitamin D deficiency in systemic lupus erythematosus (SLE) and how this may relate to fatigue and type I interferon signature, both prevalent in SLE. It appears the effect of vitamin D supplementation on fatigue and SLE disease activity is not yet clear and the jury is still out.

Efstathiou et al.^4^ reported the effect of selective Cox-2 inhibitors celecoxib, meloxicam and aceclofenac on metalloproteinases (MMP)-1, -3, and -8, and their inhibitor, TIMP-1 in 3 small groups of patients with hand osteoarthritis (OA), knee OA and hip OA, in a cross-over study design. MMP-3 was reduced after treatment with celecoxib of meloxicam in knee OA and hip OA and appears to be a sensitive biomarker in knee OA and hip OA.

Abd-Alrasool et al.^5^ studied depression in systemic lupus erythematosus (SLE) patients in Iraq. They found high frequency of depression in patients with high disease activity (SLEDAI>12) (40%) compared to patients with low/moderate disease activity (20%), and this should be kept in mind when we, as practitioners, take care of patients with SLE.

In a commentary, Cefai at al.^6^ asked the question whether rheumatologists or non-rheumatologists in Malta fare better in diagnosing and managing giant cell arteritis. They showed that temporal artery biopsy (TAB) requested by rheumatologists was more likely to be positive than TAB requested by non-rheumatologists, but the percentage was low (30% vs 14%). Also, rheumatologists appeared to adhere better to guidelines.

A case report from New York, USA^7^ describes an oxygen-dependent patient with anti-synthetase syndrome-associated interstitial lung disease who responded well and weaned off oxygen after IV pulse corticosteroids, mycophenolate mofetil and subsequent mabthera. It should be mentioned that antisynthetase syndrome is often a severe disease and prompt recognition and initiation of proper treatment regimen is critical to patient outcome.

In two cases with Still’s disease, Gao et al.^8^ used a multibiomarker disease activity test (Vectra DA) to see if they help in decisions for treatment. Vectra DA test encompasses 12 biomarkers: YKL-40 [cartilage glycoprotein 39], IL-6, leptin, CRP, SAA, MMP-1, MMP-3, resistin, vascular cell adhesion molecule (VCAM)-1, TNFRI, vascular endothelial growth factor (VEGF)-A, and epidermal growth factor (EGF). It is used in rheumatoid arthritis, where a score 45–100 denotes high disease activity. A Still’s disease patient with a score 80 did not respond to corticosteroids plus methotrexate and responded to corticosteroids plus IL-1 blockade. However, it should be stressed that Still’s disease is quite different from rheumatoid arthritis and many times carries a very high inflammatory burden.

Loukadaki et al.^9^ report on a patient with pregnancy/lactation osteoporosis and review this interesting topic. A 39-year-old woman developed severe osteoporosis after a tween pregnancy and breast-feeding her twins.

Finally, Thomas et al.^10^ in a study protocol will examine the course of herpes zoster virus (HZV) and the effect of conventional and biological disease-modifying antirheumatic drugs on HZV-specific cell-mediated immunity.
